# Pilot study to identify missed opportunities for prevention of childhood tuberculosis

**DOI:** 10.1007/s00431-022-04537-1

**Published:** 2022-06-30

**Authors:** Cornelia Feiterna-Sperling, Janine Thoulass, Renate Krüger, Walter Haas, Barbara Hauer

**Affiliations:** 1 Department of Pediatric Respiratory Medicine, Immunology and Critical Care Medicine, Charité - Universitätsmedizin Berlin, corporate member of Freie Universität Berlin and Humboldt-Universität zu Berlin, Berlin, Germany; 2 Health Protection Scotland, Glasgow, Scotland; 3 Postgraduate Training for Applied Infectious Disease Epidemiology, Robert Koch Institute, Berlin, Germany affiliated to the European Programme for Intervention Epidemiology Training, European Centre for Disease Prevention and Control (ECDC), Stockholm, Sweden; 4 Respiratory Infections Unit, Department for Infectious Disease Epidemiology, Robert Koch Institute, Berlin, Germany

**Keywords:** Childhood tuberculosis, Source case, Preventive treatment, Contact tracing

## Abstract

Tuberculosis (TB) in exposed children can be prevented with timely contact tracing and preventive treatment. This study aimed to identify potential barriers and delays in the prevention of childhood TB in a low-incidence country by assessing the management of children subsequently diagnosed with TB. A pilot retrospective cohort study included children (< 15 years) treated for TB between 2009 and 2016 at a tertiary care hospital in Berlin, Germany. Clinical data on cases and source cases, information on time points of the diagnostic work up, and preventive measures were collected and analyzed. Forty-eight children (median age 3 years [range 0.25–14]) were included; 36 had been identified through contact tracing, the majority (26; 72.2%) being < 5 years. TB source cases were mostly family members, often with advanced disease. Thirty children (83.3%) did not receive prophylactic or preventive treatment, as TB was already prevalent when first presented. Three cases developed TB despite preventive or prophylactic treatment; in three cases (all < 5 years), recommendations had not been followed. Once TB was diagnosed in source cases, referral, assessment, TB diagnosis, and treatment were initiated in most children in a timely manner with a median duration of 18 days (interquartile range 6–60, range 0–252) between diagnosis of source case and child contact (information available for 35/36; 97.2%). In some cases, notable delays in follow-up occurred.

*Conclusion*: Prompt diagnosis of adult source cases appears to be the most important challenge for childhood TB prevention. However, improvement is also needed in the management of exposed children.

**Table:** 

**What is Known:** *• Following infection with Mycobacterium tuberculosis, young children have a high risk of progression to active and severe forms of tuberculosis (TB).* *• The risk of infection and disease progression can be minimized by prompt identification of TB-exposed individuals and initiation of prophylactic or preventive treatment.*	
**What is New:** *• We could show that there are avoidable time lags in diagnosis in a relevant proportion of children with known TB exposure.* *• Delayed diagnosis of adult source cases, losses in follow-up examinations, and delay in referral to a specialized TB clinic of TB-exposed children, especially among foreign-born children, appear to be the main issue in this German pediatric study cohort.*

**What is Known:**

*• Following infection with Mycobacterium tuberculosis, young children have a high risk of progression to active and severe forms of tuberculosis (TB).*

*• The risk of infection and disease progression can be minimized by prompt identification of TB-exposed individuals and initiation of prophylactic or preventive treatment.*

**What is New:**

*• We could show that there are avoidable time lags in diagnosis in a relevant proportion of children with known TB exposure.*

*• Delayed diagnosis of adult source cases, losses in follow-up examinations, and delay in referral to a specialized TB clinic of TB-exposed children, especially among foreign-born children, appear to be the main issue in this German pediatric study cohort.*

## Introduction

There was an estimated 10 million cases of tuberculosis (TB) globally in 2019 of which 1.2 million were children [1]. Though the burden is highest in low and middle-income countries, TB remains a public health problem in high-income, low-incidence countries, particularly in vulnerable populations. Germany represents a low-incidence country, with 4791 cases and a case notification rate of 5.8 cases/100,000 population in 2019, including 196 children < 15 years of age [2].

Children, particularly those < 5 years, have the highest risk of progression to TB disease following infection [3, 4]. Progression is mostly more rapid than in adults and associated with severe forms such as miliary TB and TB meningitis [3, 5, 6]. Furthermore, TB infection in children often indicates recent transmission within the community, mainly within the same household [7].

Both the risk of initial infection and disease progression can be minimized by prompt identification of TB-exposed children and initiation of appropriate management [8, 9]. Therefore, systematic testing with subsequent treatment is recommended by the World Health Organization (WHO) and national guidelines [10–12]. National guidelines propose the use of an interferon-gamma release assay (IGRA) or tuberculin skin test (TST) for testing of TB infection; in children at high risk for disease (< 2 years of age, HIV-infection), the combination of both tests is recommended. Child contacts < 5 years with no evidence of infection with *Mycobacterium tuberculosis* at the time of examination should promptly be provided with “prophylactic” treatment for 8 weeks (preferably daily isoniazid) according to national guidelines to prevent or stop an ongoing infection. In children with a positive TST and/or IGRA without clinical or radiologic signs of TB either at initial presentation or after completion of prophylactic treatment, “preventive” treatment of latent tuberculosis infection (LTBI) should be provided [10, 13, 14]. Unless otherwise indicated by drug susceptibility testing of the source case, this consists of daily isoniazid for 9 months or daily isoniazid and rifampicin for 3 months.

Prophylactic and preventive treatment is usually well-tolerated [15] and has been shown to be very effective [8, 9]. However, evidence from high-incidence settings shows multifactorial barriers to successful implementation including factors concerning screening and adherence, healthcare provider acceptability, and fear of isoniazid resistance [16].

The aim of our pilot study was to describe retrospectively the time course and assessment steps in the management of a clinical cohort of children diagnosed with TB to identify potential barriers and delays in the prevention of childhood TB.

## Methods

### Study design

We performed a retrospective cohort study including children < 15 years diagnosed and treated for TB at the pediatric TB outpatient clinic, Charité—Universitätsmedizin Berlin, Germany, notified from January 1, 2009, to June 30, 2016, and resident in Berlin. We also included children whose first clinical contact was at a different facility before referral.

Data was extracted from the patients’ files by a specialist study nurse on selected sociodemographic details (gender, age, region of birth, parents’ region of birth, nationality), mode of case finding, diagnosis (diagnostic tests and results, date of diagnosis, primary and secondary site of disease), date of notification and initiation of treatment, drug sensitivity test (DST) result, history of exposure (timing, duration, type), and any history relating to clinical assessment (date and age at assessment, tests done, outcome). If a recognized exposure preceded the child’s TB diagnosis, data were collected on management (date of identification as a contact, date and regimen of prophylactic or preventive treatment), and the source cases’ clinical history (start of symptoms, date of diagnosis, clinical diagnosis, smear and culture result, DST result). We included details of disease site differentiated by pulmonary and extrapulmonary. Data were entered into an electronic case report form (EpiData Software, Version 4.4.3.1) and after pseudonymisation analyzed by a research scientist.

### Study definitions

A *case of TB* is defined as a child < 15 years started on TB treatment with or without laboratory confirmation (by microscopy, culture, or PCR) [2]. TB exposure is defined as a recent close contact with a person with infectious pulmonary TB [10]. A *source case* is the person with TB disease identified as the source of the childhood TB. *Mode of case finding* takes active and passive case finding into account. *Active case finding* includes contact tracing and screening for other reasons. *Passive case finding* defines identification of cases presenting for investigation of symptoms or post-mortem. *Pulmonary TB* is defined as TB of the lung parenchyma or the tracheobronchial tree; *extrapulmonary TB* is defined as TB manifestation of other organs, including intrathoracic lymphnode TB.

### Descriptive analysis

We conducted a descriptive analysis of sociodemographic, epidemiological, and clinical characteristics of the study cohort.

To identify potential differences between children with TB treated at the Charité and those treated by other health care providers, case characteristics were compared to all children notified during the study period across Berlin to the Robert Koch Institute [2].

**Fig. 1:**
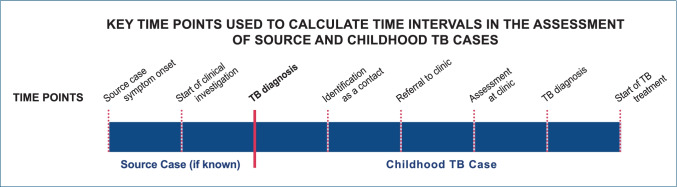
Key time points used to calculate time intervals in the assessment of source and childhood TB cases

The primary outcome measure of the study was time lags or failed steps between exposure and diagnosis of childhood TB case. This included delay or failure in identification of an infectious source case, initiation of contact investigation, referral, investigation, clinical assessment, diagnosis, and treatment initiation in the child. Time lags were calculated based on available information on key time points in the pathway (Fig. 1).

### Statistical analysis

Data were described in absolute numbers, percentages, and means (95% confidence intervals) or medians (range, interquartile range [*IQR*]) as appropriate. Differences between these factors by mode of case finding were described using the chi-squared test for categorical independent variables and the *t*-test for continuous independent variables. Fisher’s exact test was used for the analysis of group differences whose expected frequencies did not meet the statistical assumptions for the application of chi-square. All tests used were two-sided and considered significant if *p* < 0.05. All analyses were performed using STATA (version 14.1, StataCorp, LP, TX, USA) software.

### Ethical approval and data protection

The study was approved by the Ethics Committee of the Charité—Universitätsmedizin Berlin (No. EA2/183/16) and the Data Protection Office of the Robert Koch Institute for storage and analyses of study data.

## Results

### Characteristics of the study cohort

During the study period, a total of 101 childhood TB cases were notified across Berlin. Of these, 48 children were managed at the Charité and enrolled in the study. The main sociodemographic and clinical characteristics of the study population, stratified by active and passive case finding, are shown in Table 1. These were comparable to the group of all notified TB cases (including the study population, as cases could not be matched due to data protection regulations). The only difference was a higher proportion of intrathoracic lymph node disease reported as the primary disease site in the study cohort (31.3% vs. 9.9%) (Table 2).

**Table 1:** Sociodemographic and disease characteristics of the study population by mode of case finding

	Overall (*n* = 48)		Active case finding (*n* = 39)		Passive case finding (*n* = 9)		
**Study population**	*n*	%	*n*	%	*n*	%	*p*
* Gender*							0.926
Male Female	26 22	54.2 45.8	21 18	53.9 46.2	5 4	55.6 44.4	
* Age (years)*							0.615
0–4 5–9 10–14	31 13 4	64.6 27.1 8.3	26 10 3	66.7 25.6 7.7	5 3 1	55.6 33.3 11.1	
* Place of birth child*							0.232
German-born Foreign-born Missing	28 18 2	58.3 37.5 4.2	25 13 1	64.1 33.3 2.6	3 5 1	33.3 55.6 11.1	
* BCG-vaccination status (foreign-born)*							0.598
Evidence of BCG vaccination No evidence of BCG vaccination	9 9	50.0 50.0	6 7	46.2 53.8	3 2	60.0 40.0	
**Disease characteristics**							
* Primary disease site*							0.697
Pulmonary Extrapulmonary	32 16	66.7 33.3	25 14*	64.1 35.9	7 2**	77.8 22.2	
* Secondary disease site*							0.198
None Intrathoracic lymphnodes Other	40 4 4	83.3 8.3 8.3	33 4 2	84.6 10.3 5.1	7 0 2	77.8 0 22.2	
* Any extrapulmonary site*							0.0393
None/intrathoracic lymphnodes Other	43 5	89.6 10.4	37 2	94.9 5.1	6 3	66.7 33.3	(Fisher’s exact test)
**Source patient**							
* Source patient*							NA
Father Mother Sibling Grandparents Other family members Non-family members None identified	11 7 5 3 4 8 10	22.9 14.6 10.4 6.3 8.3 16.7 20.8	10 7 5 2 4 8 3	25.6 17.9 12.8 5.1 10.3 20.5 7.7	1 0 0 1 0 0 7	11.1 0.0 0.0 11.1 0.0 0.0 77.8	

*NA* not applicable, *BCG* Bacillus Calmette-Guérin

^*^Includes one case of TB meningitis

^**^Includes one case of miliary TB

**Table 2:** Comparison of study cases to all cases notified in Berlin over the study period (2009–2016)

	Charité *n* = 48		Berlin *n* = 101	
	*n*	%	*n*	%
Gender				
Male Female	26 22	54.2 45.8	64 37	63.4 36.6
Age				
0–4 5–9 10–14	31 13 4	64.6 27.1 8.3	53 27 21	52.5 26.7 20.8
Place of birth				
German-born Foreign-born Missing	28 18 2	58.3 37.5 4.2	55 46	54.5 45.5
Method of case finding				
Contact tracing Screening Passive case finding Other Missing	36 3 9 0 0	75.0 6.3 18.8 0 0	63 7 25 5 1	62.4 6.9 24.8 5.0 1.0
Primary disease site				
Pulmonary Intrathoracic lymphnodes Extrapulmonary	32 15 1	66.7 31.3 2.1	84 10 7	83.2 9.9 6.9

#### Sociodemographic characteristics of the study population

Among the children, 26 were male (54.2%), the median age was 3 years (range 0.25–14), and the majority of children were younger than 5 years (64.6%; Table 1). Twenty-eight (58.3%) children were German-born; however, in 91.7% of all cases, either patients or at least one parent was foreign-born (data not shown).

#### Site of disease

In 66.7% of children, the lung was the main affected organ. Extrapulmonary TB was the primary site of disease in 16 children (33.3%). Of those, intrathoracic lymphnode TB was present in 93.8% (15/16). Two children were diagnosed with severe disease (one TB meningitis, and one miliary TB).

#### Bacteriological confirmation and DST results

A specimen for culture was collected from 93.8% (45/48) of the children. Of these, *Mycobacterium tuberculosis* (PCR and/or culture) was detected in 31.1% (14/45). Positive cultures were documented in three out of 16 cases (18.8%) with bronchoalveolar lavage samples obtained and in 11 out of 31 cases (35.5%) with testing of gastric aspirates. DST was available for 29 children, either of their own isolate (14) and/or from the isolate of their source case (34). Most isolates (29/34; 85.3%) were fully susceptible; 4 children with culture-proven *M. tuberculosis* had drug-resistant strains (2 with isoniazid monoresistance, 1 resistant to streptomycin, and 1 to isoniazid and rifampicin).

#### IGRA and TST results

In 97.9% (*n* = 47) of the 48 childhood TB cases, TST and/or IGRA test results were documented. Of those, 78.7% (37/47) were positive using TST, 86.0% (37/43) by IGRA, and 89.4% (42/47) were positive using either or both tests at any time point (data not shown).

#### Mode of case finding

In the study cohort, the majority (81.3%) had a history of active case finding; only nine children (18.7%) had a history of passive case finding (Table 2). Of those found through passive case finding, source cases with TB symptoms (> 3 months) were identified retrospectively in two children. Of those found through active case finding, the majority (92.3%) had a history of contact tracing; three children (7.7%) were found through screening (two prior to school entry, one during an asylum-seeking process; for these children, no source case could be identified). In the group of children diagnosed through active case finding, 10 out of 39 (25.6%) had signs or symptoms consistent with TB disease.

There were no significant differences in available sociodemographic characteristics by mode of case finding (Table 1) nor in the proportion of children with pulmonary disease (64.1% [25/39] vs. 77.8% [7/9]; *p* = 0.697). However, statistical analysis confirmed a significantly lower proportion of extrapulmonary manifestation of TB (apart from intrathoracic lymphnodes as manifestation site) for children who were identified through active case finding compared to children identified through passive case finding (5.1% vs. 33.3%; *p* = 0.0393, Fisher’s exact test).

### Contact tracing and exposure

A total of 36 children were diagnosed through contact tracing. Twenty-six of these children (72.2%) were < 5 years. In the majority of cases, the source was a family member including any of the parents (17; 47.2%), siblings (5; 13.9%), grandparents (2; 5.6%), and other family members (4; 11.1%). Non-family members included family friends (4; 11.1%), child carers (3; 8.3%), or others (1; 2.8%) (Table 2). Additional information on the duration of exposure was available for 41.7% (15/36), showing a median exposure of 3 months (*IQR* 2–4; range 0.5–12).

#### Time lags in the pathway

**Fig. 2:**
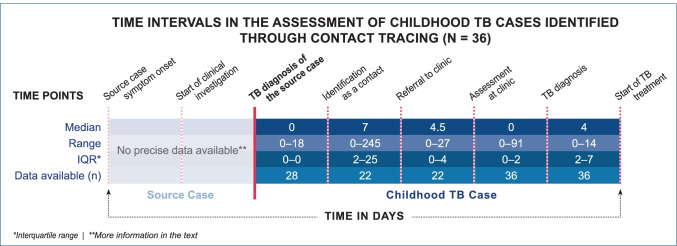
Key intervals in the assessment of childhood TB cases identified through contact tracing (*N* = 36)

Time intervals with potential lags—from first symptoms of the source case to start of TB treatment in affected children—are depicted in Fig. 2. For source cases, there was limited information available on precise time intervals between the onset of first symptoms and first clinical investigation. All had been diagnosed with pulmonary TB. In 86.1% (31/36) of the sources, it was explicitly stated that the pulmonary TB was infectious; 23 of them were sputum smear positive, and one case was diagnosed post-mortem. In four source cases, disease features were consistent with advanced disease (cavitations or requiring ventilation). Overall, 67.6% (23/34) of source cases had either a history of at least 3 months of symptoms or features reported consistent with severe disease.

Median time between diagnosis of the source case and identification of the child through contact tracing was 0 days (*IQR* 0–0, range 0–18; information available for 77.8% [28/36]).

Formal referral dates were available for 61.1% (22/36) of children, showing a median of 7 days (range 0–245) between start of contact tracing and referral and a median of 4.5 days (range 0–27) from referral to clinical assessment. In 25% (9/36) of children, any TB testing was performed prior to the date of formal referral.

Median time between identification of the child as a contact and clinical assessment at the TB clinic was 17 days (range 2–252).

Median time between diagnosis of the source case and any first assessment (Charité or any other health facility) of the child contact was 8 days (*IQR* 4–27; range 0–66; data available for 97.2% [35/36]). Overall, 65.7% (23/35) of children were assessed within 14 days after TB diagnosis of the source case.

Median time between first clinical assessment and diagnosis of childhood TB was 0 days (range 0–91), and median time between TB diagnosis and start of TB treatment was 4 days (range 0–14).

There was sufficient information to evaluate the total time between diagnosis of the source case and the diagnosis of the child contact in 97.2% (35/36) with a median duration of 18 days (*IQR* 6–60, range 0–252).

Overall, 30.6% of the children (11/36) were diagnosed within 1 week after the source patient had been diagnosed; this proportion was higher for German-born compared to foreign-born patients (39.1% vs. 9.1%; *p* = 0.072). By 2 and 3 completed weeks, 47.2% (17/36) and 61.1% (22/36) cases had been diagnosed, respectively (German vs. foreign-born 56.5% vs. 27.3%; *p* = 0.15 and 65.2% vs. 54.5%; *p* = 0.55).

#### Preventive measures

Thirty of the 36 cases (83.3%) had not received prophylactic or preventive treatment, as they had already prevalent TB disease when first presented. Three out of 26 children < 5 years did not receive prophylactic treatment for unknown reasons, and another two children developed TB disease despite prophylactic treatment. One child > 5 years of age was diagnosed with LTBI (3 months after exposure) and had developed TB at the end of preventive treatment (3 months isoniazid plus rifampicin).

## Discussion

Our pilot study provides data of a cohort of 48 childhood TB cases. Thirty-six children had been identified through contact tracing, the majority being < 5 years. Children were mostly infected by family members. In 67.6% (23/34) of source cases, there was either a history of at least 3 months of symptoms or there were features reported consistent with severe disease.

Thirty out of 36 children already suffered from TB disease at the time of first clinic evaluation. Despite a short time lag of a median of 17 days from identification of the source case to presentation of the child in our clinic, it was still too late to initiate prophylactic or preventive treatment. The overrepresentation of young children < 5 years reflects the higher risk of infection and more rapid disease progression in this age group and stresses the importance of contact tracing [17].

Data from low-incidence countries on implementation of preventive measures for children are scarce. A study in the USA found that 40% of children < 5 years with TB disease detected through contact tracing had delayed or failed steps in their investigation or management [18]. A second US study identified shortcomings in 16% of children < 14 years [19]. An Australian study described notable losses in TB contact tracing for children < 5 years around referral to TB clinics [20]. A study from Germany showed that in children (*n* = 276) up to the age of 5 years identified through contact tracing, only 32% were screened according to current guidelines and only 20% received prophylactic and/or preventive treatment [21].

Our findings are consistent with a recently published meta-analysis [17], showing that most cases of childhood TB occurred within weeks of contact investigation initiation and were therefore hardly preventable through prophylaxis.

Hilar lymphadenopathy, a radiologic sign of an early stage of disease [22], was described in the majority of our study cohort. The study population appeared to be similar to the wider Berlin pediatric population diagnosed with TB with the exception of a higher proportion of intrathoracic lymphnode disease. We hypothesize that in settings being not familiar with the specific disease patterns of childhood TB, hilar lymphadenopathy may potentially be misclassified as pulmonary TB.

The proportions of children with pulmonary TB as primary diagnosis did not differ by mode of case finding. However, children found through passive case finding were more often diagnosed with non-intrathoracic lymphnode TB, possibly reflecting the diagnostic delay [3].

From a clinical point of view, we observed no critical time lags between first presentation of children, diagnosis, and treatment initiation. Therefore, we can speculate that prompt treatment initiation prevented more severe disease forms. The proportion of German-born cases found through active case finding was almost twice as high as in foreign-born cases (though high proportions of German-born children had at least one foreign-born parent). Although not statistically significant, these findings are consistent with prior analysis of national notification data [23]. In our study, foreign-born children were less likely to be diagnosed at 1, 2, and 3 weeks after source case identification.

Other studies demonstrated that foreign-born TB contacts are less likely to start preventive treatment [24–26] and are at higher risk of developing TB disease [24, 27]. These and our own findings may be influenced by factors such as language skills and better access to health care in families living in Germany for a longer time, but this warrants further and larger studies.

Less than a third of the cases were bacteriologically confirmed, proving that bacteriological confirmation is difficult due to paucibacillary disease in children [28]. Where information on DST was available, the majority of cases (85.3%) were classified as fully sensitive TB. TST and/or IGRA was false-negative in 10.6% of the cases, underscoring the importance of a careful clinical assessment and reading of radiological findings in childhood TB contacts, irrespective of TST and IGRA results.

Exposure was largely reported within households or to other very close contacts. Once the source case was diagnosed, children were identified quite promptly. However, nearly three quarters of source cases presented with either a quite advanced stage or prolonged course of disease (median 3 months). Such an exposure was also identified in two children found by passive case finding. It is well-documented that prolonged exposure and high bacterial load are associated with increased risk of TB transmission [5]. We presume that TB could have been prevented in many exposed children through timely identification of the source case. Delayed diagnosis of pulmonary TB is frequently reported in high TB–incidence low- and middle-income countries [29], but also in low-incidence countries [30, 31]. Furthermore, transmission prior to TB symptoms in subclinical source cases may occur [32]; thus, documented exposure times may be underestimated.

In few children, exceptionally long time periods between identification and referral or start of clinical assessment were observed because they presented first at different health care facilities and missed follow-up appointments or were first diagnosed with LTBI and subsequently developed TB at the end of a prophylactic and preventive treatment.

There exist no standards in terms of recommended contact tracing time frames in Germany. In a Californian study, a maximum of 14 days between the reporting of a source case and TST in childhood contacts was recommended [18].

Our study has some limitations: first, this was a pilot study with small case numbers. Second, it was a retrospective analysis based on patient records. Many of the variables were extracted from case notes, and the information documented there may not always have been collected systematically or in a standardized way. We experienced some missing data, which is often a limitation of retrospective folder reviews. Variation in completeness may also be partly due to documentation changes over time. We experienced improvements in data completion in the case files over time, partly due to enhanced information exchange, both with the referring institutions and responsible public health offices.

Furthermore, validity of some information provided by the parents at the time of admission may have been potentially impacted by patient or parent recall.

Our pilot study demonstrates the importance of a thorough and systematic routine data documentation and collection, both on TB source cases and exposed children, allowing future operational research to optimize TB prevention and minimize delays for children exposed to TB in Germany.

## Conclusion

This pilot study reveals that there are potentially avoidable time lags in the assessment of TB-exposed children. Delayed diagnosis of adult source cases, losses in follow-up examinations, and delay in referral to a specialized TB clinic have to be avoided, as rapid identification of exposed children is mandatory due to the high risk of infection and disease progression. Guidance on the length of individual steps in the process together with improved monitoring could contribute to improvements in childhood TB prevention and control.

## Data Availability

The datasets generated during and analyzed during the current study are available from the corresponding author on request in accordance with data protection requirements.

## Abbreviations

DST: Drug sensitivity testing
IQR: Interquartile range
IGRA: Interferon-gamma release assay
TB: Tuberculosis
TST: Tuberculin skin test
WHO: World Health Organization

## Code availability

EpiData Software, Version 4.4.3.1; STATA (version 14.1, StataCorp, LP, TX, USA) software.
