# Correction to: Pilot study to identify missed opportunities for prevention of childhood tuberculosis

**DOI:** 10.1007/s00431-022-04571-z

**Published:** 2022-07-28

**Authors:** Cornelia Feiterna-Sperling, Janine Thoulass, Renate Krüger, Walter Haas, Barbara Hauer

**Affiliations:** 1 Department of Pediatric Respiratory Medicine, Immunology and Critical Care Medicine, Charité - Universitätsmedizin Berlin, Corporate member of Freie Universität Berlin and Humboldt-Universität zu Berlin, Berlin, Germany; 2 Health Protection Scotland, Glasgow, Scotland; 3 Postgraduate Training for Applied Infectious Disease Epidemiology, Robert Koch Institute, Berlin, Germany affiliated to the European Programme for Intervention Epidemiology Training, European Centre for Disease Prevention and Control (ECDC), Stockholm, Sweden; 4 Respiratory Infections Unit, Department for Infectious Disease Epidemiology, Robert Koch Institute, Berlin, Germany

## Correction to: European Journal of Pediatrics 10.1007/s00431-022-04537-1

In the Table 1 of the original published version of this article, the table entry "20.8" in Overall (n = 48) under % column should have been presented along with None identified under Source patient section. The corrected Table 1 is shown as follows.

The original article has been corrected.

**Table 1:** Sociodemographic and disease characteristics of the study population by mode of case fnding

	Overall (*n* = 48)		Active case finding (*n* = 39)		Passive case finding (*n* = 9)		
**Study population**	*n*	*%*	*n*	*%*	*n*	*%*	*p*
* Gender*							0.926
Male	26	54.2	21	53.9	5	55.6	
Female	22	45.8	18	46.2	4	44.4	
* Age (years)*							0.615
0–4	31	64.6	26	66.7	5	55.6	
5–9	13	27.1	10	25.6	3	33.3	
10–14	4	8.3	3	7.7	1	11.1	
* Place of birth child*							0.232
German-born	28	58.3	25	64.1	3	33.3	
Foreign-born	18	37.5	13	33.3	5	55.6	
Missing	2	4.2	1	2.6	1	11.1	
* BCG-vaccination status (foreign-born)*							0.598
Evidence of BCG vaccination	9	50	6	46.2	3	60	
No evidence of BCG vaccination	9	50	7	53.8	2	40	
**Disease characteristics**							
* Primary disease site*							0.697
Pulmonary	32	66.7	25	64.1	7	77.8	
Extrapulmonary	16	33.3	14*	35.9	2**	22.2	
* Secondary disease site*							0.198
None	40	83.3	33	84.6	7	77.8	
Intrathoracic lymphnodes	4	8.3	4	10.3	0	0	
Other	4	8.3	2	5.1	2	22.2	
* Any extrapulmonary site*							0.0393
None/intrathoracic lymphnodes	43	89.6	37	94.9	6	66.7	(Fisher’s exact test)
Other	5	10.4	2	5.1	3	33.3	
**Source patient**							
* Source patient*							NA
Father	11	22.9	10	25.6	1	11.1	
Mother	7	14.6	7	17.9	0	0	
Sibling	5	10.4	5	12.8	0	0	
Grandparents	3	6.3	2	5.1	1	11.1	
Other family members	4	8.3	4	10.3	0	0	
Non-family members	8	16.7	8	20.5	0	0	
None identified	10	20.8	3	7.7	7	77.8	

*NA* not applicable, *BCG* Bacillus Calmette-Guérin

*Includes one case of TB meningitis

**Includes one case of miliary TB

